# Clinical Evaluation of a Novel Technology for Oral Patient-Controlled Analgesia, the* PCoA® Acute* Device, for Hospitalized Patients with Postoperative Pain, in Pilot Feasibility Study

**DOI:** 10.1155/2017/7962135

**Published:** 2017-09-28

**Authors:** Stefan Wirz, Stefan Conrad, Ronit Shtrichman, Kai Schimo, Eva Hoffmann

**Affiliations:** ^1^Department of Anesthesia, Intensive Medicine, Pain Medicine/Palliative Medicine, Center for Pain Medicine, CURA Hospital GFO, Bad Honnef, Germany; ^2^DosentRx Ltd., 2 Hahar St., Har Tuv Industrial Zone A, 9980101 Har Tuv, Israel; ^3^Department of Anesthesia, Interdisciplinary Intensive Care, Emergency Medicine and Pain Medicine, EvK-Protestant Hospital Herne, Herne, Germany

## Abstract

**Background:**

Acute postoperative pain delays recovery and increases morbidity and mortality. Traditional administration of postoperative analgesics by nurses is often inefficient. The present study evaluated the safety, efficacy, and usability of a novel, patient-controlled analgesic dispenser, the PCoA Acute.

**Methods:**

A controlled pilot study was conducted at three medical centers. Patients scheduled for elective surgery were enrolled into two groups, both taking oral analgesics: a control group (*n* = 43), opioids dispensed by nurses, and a test group (*n* = 27), opioids dispensed via the PCoA Acute. Pill intake data were recorded. Pain ratings at rest and during movement were surveyed.

**Results:**

No severe adverse events were recorded. Average pill intake time was reduced from 8 : 58 minutes in the control group to 1 : 17 minutes in the test group (*P* value < 0.05). The test group took 67% more pills than the control group, indicating enhanced compliance. Pain scores were significantly lower for patients in the test group (*P* value < 0.05). Over 90% of PCoA Acute users were satisfied with its use.

**Conclusions:**

The study confirmed that PCoA Acute is safe and effective. It is well accepted by patients and medical staff. Its use can optimize pain medication administration.

## 1. Introduction

The management of acute pain in the hospital setting is a continuing challenge for healthcare professionals. More than 80% of patients who undergo surgical procedures experience acute postoperative pain. Most of them report inadequate pain relief, leading to increased morbidity, mortality, and costs [1]. Prevention and effective relief of acute pain may improve clinical outcomes, avoid clinical complications, save health care resources, and improve quality of life [2].

In most hospitals nurses play a key role in the assessment and management of patients' pain. However, it has been shown that nurses tend to underestimate patients' pain and undermedicate patients for their pain [3]. Other factors in poor pain management include fear of complications associated with analgesic drugs and inadequate nurse staffing. The mainstay of postoperative pain therapy is opioids, which have significant side effects, and their long-term use can lead to dependence and addiction [2]. Consequently, nurse provision of analgesics to the patient is a strictly controlled and time-consuming procedure [4], and the burden on nursing staff administering pain medications encumbers a significant portion of their working time [5].

Patient-controlled analgesia (PCA) is a delivery system in which patients self-administer predetermined doses of analgesic medication to relieve their pain. PCA has become a standard of care in pain management. However, most common routes of PCA administration are intravenous (IV) and epidural (PCEA) [6]. Advantages of PCA over traditional analgesics administration include better pain control and greater patient satisfaction. When compared with nurse-controlled analgesia, the PCA technique has demonstrated better analgesic effects, decreased patient anxiety, less sedation, and possibly fewer postoperative complications [7, 8]. PCA also benefits patients indirectly by alleviating the time demand on nurses [9]. However, the limitations of IV PCA and PCEA, mostly related to their invasiveness, include operator errors and pump malfunction and infection. Moreover, the PCA device must be monitored frequently to prevent tampering and patient mobility is limited by the pump. Therefore, IV PCA is mainly suitable for patients with background infusion [6, 10]. Hence, the oral route of postoperative analgesics is strongly recommended for patients who can use it [11]. Several oral PCA devices have been evaluated in recent years and found to be safe and effective for provision of postoperative pain medication in the clinical setting [12, 13].

PCoA Acute is an innovative personalized oral PCA device, which provides a comprehensive solution for the provision of pain medication at the bedside. The system has the following capabilities and benefits:

- It uses pain medication in the original packaging. The device is therefore easily integrated into clinical routine.
- It safely delivers and tracks each pill right to the patient's mouth. The device verifies consumption of pills by the patient, enables stringent control of pill consumption, and monitors drug dispensing by caregivers in real time.
- It provides a locked safe for high risk narcotic drugs.
- It dispenses to the prescribed patient only, using personalized identification.
- It allows remote monitoring and management including alerts and reminders.
- It enables data collection and management related to the patient's clinical status.

**Figure 1:**
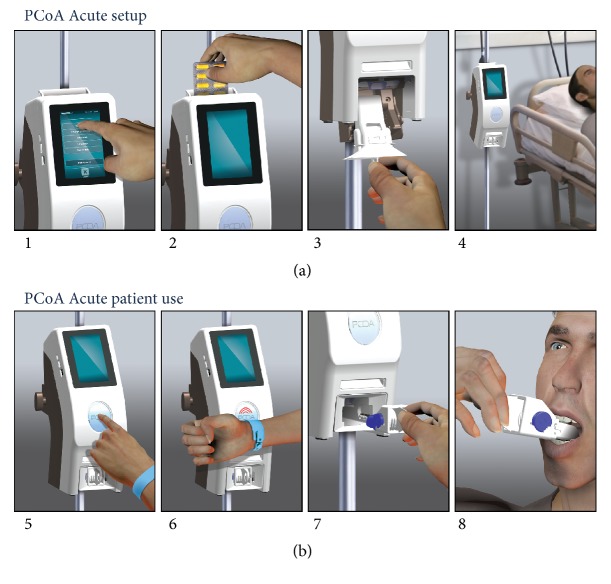
PCoA Acute device application. (a) Device setup by the nurse. (1) Patient's prescription is programmed into the device and displayed on the touch screen. (2) Drug blister pack is inserted into the Drug Dispensing Unit (DDU). (3) Disposable PillBox with integral mouth piece is inserted in the device. (4) The device is located near the patient's bedside. (b) Patient use. (5) Patient requests pill when needed by pushing a button. (6) Patient identity is confirmed by registration of RFID wristband. (7) Patient withdraws the PillBox. (8) Patient receives pill by application of light sucking pressure on the PillBox mouthpiece.

Figure 1 demonstrates the use of the PCoA Acute in the hospital setting. The PCoA Acute system comprises the following components: (1) Drug Dispensing Unit (DDU) is a safe storage container which can be opened only with a security code. The DDU is loaded at the start of therapy with a full drug blister pack. The DDU dispenses medication directly from the original packaging. This facilitates logistical control over the prescribed medication, maintains the clinical standard of care, and enables reuse of unused drugs while maintaining drug quality. The device is located by the patient's bedside and records all pill administrations to the patient. (2) A Radio Frequency Identification (RFID) wristband is used for patient's registration. (3) PillBox is a patient-specific, mouth-actuated, disposable receptacle, from which the patient receives the pill. The PillBox releases a pill only when subject to negative sucking pressure, which delivers the pill onto the patient's tongue. This technology allows stringent control over the analgesic drugs consumed by the patient.

A feasibility, pilot clinical study was conducted to initially evaluate the PCoA Acute in the clinical setting for pain medication provision to postoperative patients. To our knowledge, this is the first clinical study evaluating pain medication provision where a standard solid pill is sucked by the patient and the pill is monitored right to the patient's mouth.

The study aimed to evaluate safety, efficacy, and usability of PCoA Acute, along with the acceptance of the novel pill sucking approach by patients and medical staff. The study also aimed to demonstrate optimization of the administration process by the device, compared to the conventional method of nurse administration of pain medication.

The study specific objectives were as follows:

1. PCoA Acute safety: no pill overdose or pill malformation occurs upon dispensing and no pill inhalation occurs during pill sucking via the PillBox.
2. PCoA Acute efficacy: 90% of pill intake attempts are successful and no device critical malfunction occurs.
3. PCoA Acute usability: 90% of patients and medical staff can easily operate the device and are satisfied with its use.
4. PCoA Acute advantage over conventional method of nurse-provided medications: the time from pill request to pill intake is reduced by at least 50%.

## 2. Methods

### 2.1. The Clinical Study Design

An open-label, multicenter, controlled, feasibility pilot clinical study was conducted. The study was registered on ClinicalTrials.gov (NCT03134001). Centralized Institutional Review Board (IRB) approval of the study was given by the Ethics Committee, North Rhine-Westphalia (Düsseldorf, Germany). Subsequently, patients 18 years and older, scheduled to undergo elective surgery, provided written informed consent and were enrolled to the study. The study included a control group of 43 patients requiring postoperative pain therapy from nursing staff by conventional means. The test group comprised 27 patients who used PCoA Acute to receive their pain medication. The follow-up time was 48 hr (2 postoperative (PO) days). The time duration from pill request to pill intake by the patient was recorded as well as the number of pills obtained by each patient. Pain scores (pain at rest and during movement) were recorded by the PCoA Acute on each occasion of a pill intake. Safety and efficacy parameters of the PCoA Acute were evaluated using questionnaires filled in by patients and medical staff.

### 2.2. Participants

The study population comprised males and females, aged 18–80 and scheduled for elective operation in ENT (Ear, Nose, and Throat), Gynecology, Orthopedics, and Thoracic, and required postoperative pain therapy for at least 48 hr. All patients enrolled signed an informed consent form prior to surgery. Pain medications were identical for patients in both groups, undergoing similar surgery type in the same medical center, but varied between medical centers and surgery types (Table 1). 

*Inclusion Criteria*

1. Operative procedure with at least 3 days' hospital stay
2. Planned postoperative pain therapy with oral medication using a strong opioid
3. No contraindication for opioid therapy
4. No contraindication for oral pain therapy
5. Patient being able to understand and complete the questionnaire
6. Patient signing an informed consent form

*Exclusion Criteria*

1. Opioid or drug addiction
2. Opioid intolerance
3. Pain therapy using anesthesia, IV PCA, or infusion
4. Patient's refusal of an opioid therapy
5. Inability to swallow medicine

#### 2.2.1. Study Setting and Location

The study was conducted at three medical centers in Germany:

- Department of Anesthetics, Interdisciplinary Intensive Care, CURA Hospital, Bad Honnef—Center for Pain Medicine.
- Clinic of Anesthetics and Intensive Care, Bethanien Hospital, Moers.
- Clinic of Anesthesiology, Intensive Care and Pain Therapy, Teaching Hospital of the University of Cologne, Oberberg Hospital, Gummersbach.

#### 2.2.2. Medication for Postoperative Pain

In all 3 medical centers, there existed a standardized postoperative pain procedure involving different step 1 and step 3 analgesics. In both patient groups, basic pain medication consisting of oral nonopioid analgesics was given. Dipyrone—which is widely used in Germany—was prescribed in combination with Etoricoxib, Ibuprofen, or Diclofenac. The centers differed in the prescription of either Etoricoxib, Ibuprofen, or Diclofenac. Contraindications for the use of these step 1 analgesics were allergy, blood count abnormalities, cardiovascular diseases, renal impairment, pulmonary diseases, and any kind of gastrointestinal disorder. The daily doses for these analgesics were standardized as follows: Dipyrone: 4 × 1 g, Etoricoxib: 1 × 60 or 90 mg, Ibuprofen: 3 × 500 mg, and Diclofenac: 2 × 75 mg. With regard to the opioids, the centers used 2 different step 3 opioids: oxycodone and morphine. In addition to the “basic” treatment with step 1 analgesics, controlled-release formulation of oxycodone/naloxone is used at daily doses of 2 × 5/2,5 mg or 10/5 mg and the controlled-release formulation of morphine is used at daily doses of 2 × 10 mg. Morphine and oxycodone are *μ*-opioid receptor agonists. Oxycodone reveals binding properties at the opioid *κ*(2b) receptor. The efficacy and safety of morphine and oxycodone for postoperative pain therapy has been reported previously [11, 14].

### 2.3. Intervention

#### 2.3.1. The Test Group: Pain Medication Provision by PCoA Acute

The PCoA Acute is an oral PCA device which provides patient-controlled analgesics at the bedside. A standard drug package is inserted into the device, which is operated by a touch screen. The medication used in this study consisted of short-lasting formulation of oxycodone (5 or 10 mg) or morphine (10 mg). Medication is dispensed only after the patient's identity has been confirmed by the RFID wristband. The system connects to the hospital database. Medication history, therapies, and pain scores are saved and easily displayed on the touch screen.

Setup included device programming by a trained nurse, with the appropriate therapy regimen for each patient and loading the blister pack of the relevant pills. Patient training was then conducted by the nurse. The setup time and patient's training time were measured, for each patient, by a third person. During the study, patients used the PCoA Acute to obtain their pain medication by pressing the device's button followed by wristband registration. If the pill request was made during the safe time interval (time period in which the patient is allowed to receive medication according to the therapy regimen), the patient received a pill. However, if a pill was requested during the lockout time interval (time period in which the device is locked to prevent drug overdose) no pill was released. The device screen indicated the time left to the next pill intake. Before each pill intake, the patient was requested by the device's screen to score his pain at rest and during movement. All data were recorded and saved in the device's log files. The patient could also ask for an extra pill from the nurse, if needed. Using a security code, the nurse was able to release an extra pill during the lockout interval, subject to physician's approval. At the study's termination questionnaires were filled out by patients and nurses and the data recorded by the device were analyzed.

#### 2.3.2. The Control Group: Pain Medication Provision by Nurse

Nurse administration of pain medication, upon patient's request and according to patient's therapy regimen, is a routine procedure in hospitals. This procedure includes the following steps, conducted near the patient's bedside and also in the nurses station: a patient who feels acute pain calls the nurse and waits for her attendance. The nurse reviews the patient's prescription and time of previous drug intake. Once a new pain medication intake is approved, the nurse retrieves the key for the drug safe, dispenses the medication, and documents drug withdrawal. The nurse then returns to the patient's bedside, provides the medication, and documents the fact in the patient's record file.

**Figure 2:**
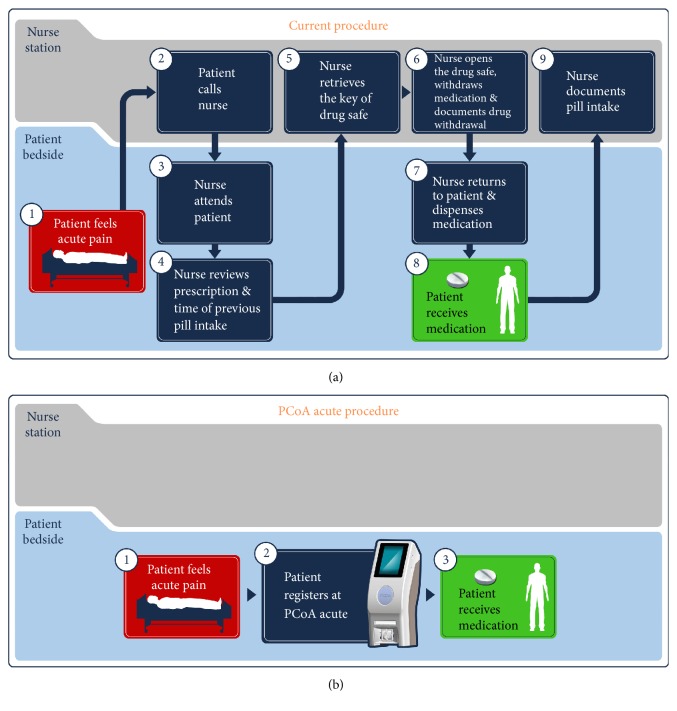
Comparison of pill provision processes evaluated in the study. (a) Current procedure involves the following steps. The patient feels acute pain (1). The patient calls the nurse (2) and waits until the nurse's attendance (3). The nurse reviews the patient's prescription and time of previous drug intake (4). Once new pain medication intake is approved, the nurse goes to the nurse station and retrieves the drug safe key (5). The nurse opens the drug safe, dispenses the medication, and documents drug withdrawal (6). The nurse returns to the patient's bedside and provides the medication (7). Only then the patient receives the medication (8) and the nurse documents pill intake in the patient's record file, located at the nurse station (9). (b) PCoA Acute process. The device is located near the patient's bedside and the procedure comprises only 3 steps: the patient feels acute pain (1). He is registered at the device (by the RFID wristband) (2) and can immediately receive his medication, subject to therapy restrictions (3).

The patients of the control group were advised to report pain to the nurses early and to order the acute pain medication. In the control group, nurses were instructed to dispense the short-lasting formulation of oxycodone (5 or 10 mg pill) or morphine (10 mg pill) to patients who reported a NRS (pain) > 3. In both groups, the acute pain medication was restricted to the short-lasting formulations of oxycodone or morphine. Nurses were informed that the time of analgesic pills administration was being recorded. During the study, a third person measured the time from a patient requesting a pill from a nurse until pill intake by the patient. Endpoint of this was the number of pills obtained by each patient which was counted by a third person and by the nurse.  Figure 2 illustrates the two processes for pain medication provision compared in the study.

### 2.4. Outcomes

The study outcomes are as follows: 

*PCoA Acute Safety*

1. No pill overdose in dispensing (e.g., two pills dispensed together, dispensing within lockout time)
2. No pills malformation upon dispensing
3. No pill inhalation during pill sucking
4. No severe adverse events related to pill intake by the PCoA Acute

The safety outcomes were measured using questionnaires filled by patients and medical staff participating in the study and data recorded by PCoA Acute devices. 

*PCoA Acute Efficacy*

1. Success rate of 90% for pill intake upon patient's request. This outcome was measured by analysis of data recorded by the device for each patient as well as by questionnaires filled out by patients.
2. No critical device malfunctions. This outcome was measured by the questionnaires filled out by patients and medical staff.
3. Time of pill intake reduced by at least 50% in the test group compared to the control group.

*PCoA Acute Usability*

1. At least 80% of patients and medical staff are satisfied with device use and will recommend its use for their colleagues. This outcome was measured using questionnaires filled out by patients and medical staff participating in the study.

### 2.5. Sample Size

The calculation of the sample size was based on demonstrating an effect size of at least 1.0 with 80% power and 5% statistical significance. Effect size is calculated as the difference in outcome between the study groups divided by the common standard deviation. A sample size of minimum 20 subjects in each group will have 80% power to detect an effect size of 1.0 using a two-group *t*-test with a 0.05 two-sided significance level [15].

### 2.6. Patients Allocation to the Study's Groups

The study had a sequential design; the control group study was conducted before initiation of the test group study. The rationale for this design is that the same nurses had a role in both groups. This design minimized nurses burden and bias towards one group. This patients' allocation approach provides random enrollment of patients to groups, by their operation date, without any selection bias. Subjects eligible for the study were assigned as follows: Bad Honnef enrollment dates: control group 16/05/2015–22/06/2015 and test group 18/08/15–24/08/15; Moers enrollment dates: control group 16/05/15–22/06/15 and test group 18/08/2015–22/06/15; Gummersbach enrollment dates: control group 03/09/2015–14/09/15 and test group 15/09/15–28/10/15.

### 2.7. Statistical Analysis

The two-sample nonparametric Wilcoxon-Mann–Whitney Rank sum test for independent samples was used to analyze the difference in outcomes between the study groups.

Analysis of variance (ANOVA) was applied for analyzing the difference in the study outcomes between study medical centers within study groups [16].

## 3. Results

### 3.1. Patient Disposition

**Figure 3:**
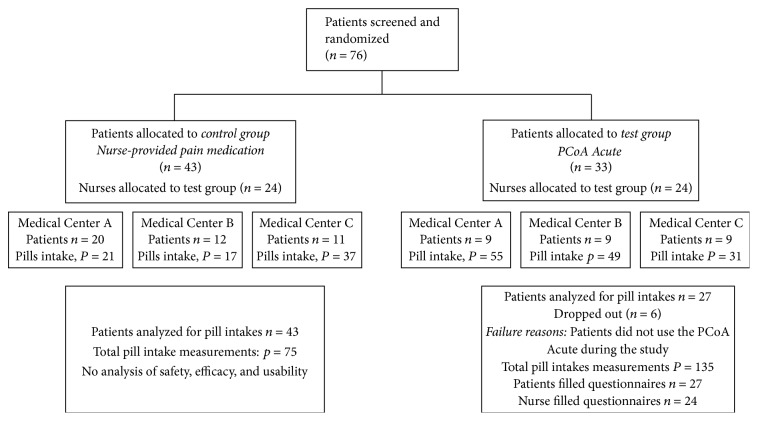
Patients disposition flow diagram.

The study was conducted at three German medical centers. The first patient was enrolled in May 2015 and the last patient was enrolled in October 2015. The whole study duration was 6 months. The patients disposition flow diagram is illustrated in Figure 3. The control group followed the conventional and routine procedure of patients receiving postoperative analgesics by nurse upon request. A total of 43 patients were enrolled to the control group and signed informed consent. Their pill intake times and the number of pills taken were measured during the study. 33 patients were initially enrolled to the test group. However, only 27 patients completed the study, used the PCoA Acute for their pill intake, and filled the questionnaire. 6 patients were excluded because they did not use the PCoA Acute during the study. Table 1 presents a summary of the demographic and baseline characteristics of the study population. 24 nurses were allocated to the study. Nurse tasks for the control group were to provide analgesics to patients upon request, while the time required for pill provision is measured. Nurse tasks for the test group were to set up PCoA Acute, train patients how to use the device, provide an extra pill from the device upon patient's request, use a secret code, and fill out the questionnaires.

### 3.2. Safety Outcomes Results

**Figure 4:**
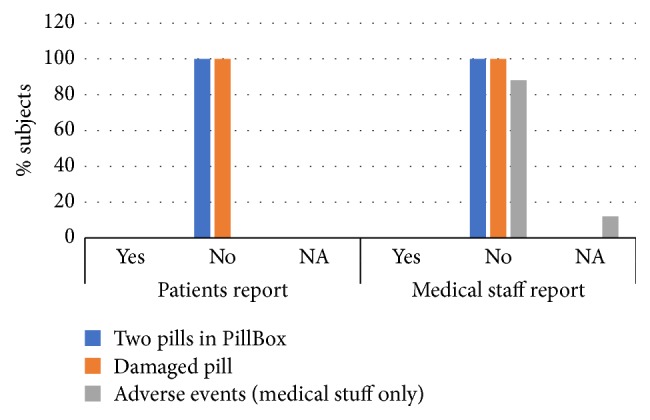
Summary of safety parameters of PCoA Acute. Both patients and medical staff were asked about occasions of 2 pills or damaged pill in PillBox. Only medical staff were asked for occasions of adverse events. Data was obtained from patients and medical staff questionnaires. Data from the PCoA Acute log files was used to confirm answers. NA: not applicable.

Safety of the PCoA Acute was evaluated by questionnaires filled in by patients and medical staff and confirmed by data recorded by the device. No incidence of pill overdose dispensed occurred and no pill malformation was reported either by patients or by medical staff. Furthermore, no severe adverse events, such as pill inhalation, were reported by medical staff (Figure 4). Based on PCoA Acute log files, no pills were dispensed within the safety lockout interval and all patients attempts to obtain a pill during the lockout time failed (data not shown).

### 3.3. Efficacy Outcomes Results

**Figure 5:**
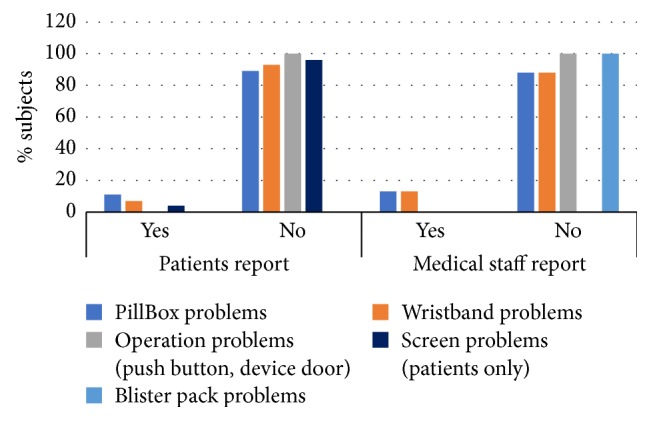
Summary of functional parameters of PCoA Acute. Both patients and medical staff were asked about problems encountered with the device operation, the PillBox, wristband, and screen. Only medical staff were asked for problems with insertion of blister pack. Data was obtained from patients and medical staff questionnaires and from the PCoA Acute log files.

Efficacy of PCoA Acute was measured by the success rate of pill intake upon patient's request. Other functions documented were attempts of pill request during the lockout interval and extra pill obtained from the nurse. The performed actions are summarized in Table 2. All test group patients (100%) successfully obtained pills according to their therapy regimen. All attempts to obtain a pill during the lockout interval failed. Such failed attempts were recorded for 60% of patients. 29% of patients used the option of extra pill provision by the nurse. Over 80% of patients and medical staff did not encounter any problem related to device functionality, including problems in the PillBox operation (e.g., extracting a pill from the PillBox); wristband registration; device malfunctions (problems with button, door, screen, etc.), and pill packaging insertion and ejection (Figure 5).

The study aimed to compare two processes of pain medication provision, use of PCoA Acute and nurse-provided medication. The duration from a patient requesting pain medication to actually receiving the pill was compared.

Table 3 shows data of time measured for pill intake in both groups and in each center. *P* value was calculated separately to distinguish differences between groups for all medical centers and within each center. All differences between groups were statistically significant. Differences observed between control groups within medical centers were not statistically significant. Differences observed between study groups of all centers were statistically significant.

Data analysis revealed that the mean time of the control group was 8.58 minutes and the maximal time was 1 hour (after 1 hr, time measurement was stopped). In contrast, the mean time to receive a pill by PCoA Acute was 1.17 minutes, including setup and training time. The maximal time was 3.77 minutes. These results indicate 86% reduction in time required to obtain a pill upon patient's request when using PCoA Acute.

Table 4 demonstrates the number of pills taken by patients in both groups. Data analysis shows that patients in the test group received a mean of 5 pills during the study, while patients in the control group received a mean of 1.67 pills. Hence, patients in the test group received on average 3 times more pills than patients in the control group.

*P* value was calculated separately to distinguish between groups for all medical centers and within each center. Differences observed between control and test groups of all centers were found to be statistically significant. Differences in medical centers A and B were found to be statistically significant, whereas differences in medical center C were not found to be statistically significant.

### 3.4. Pain Scores Recorded by Patients Using the PCoA Acute

**Figure 6:**
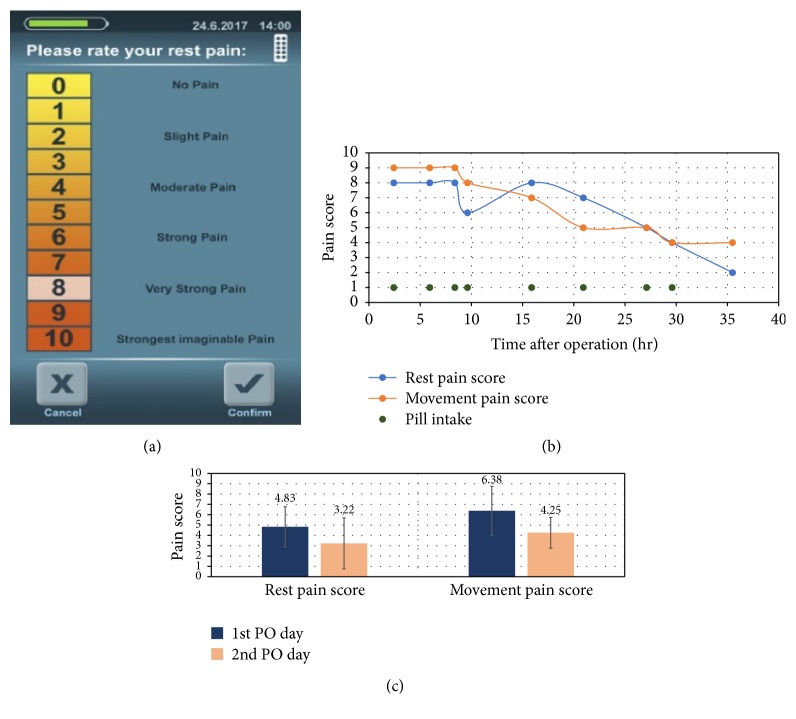
Pain scores recorded by PCoA Acute system, obtained by patients in the test group. (a) PCoA Acute survey's touch screen, demonstrating rest pain scoring (similar screen is that used to score movement pain). (b) Example of a selected patient with representative data of rest and movement pain scores obtained at each pill intake event. (c) Pain scores data analysis of patients in the test group (*N* = 18, who scored their pain at pill intake), demonstrating statistically significant pain reduction from the 1st postoperative (PO) day to the 2nd.

The PCoA Acute collects patient clinical data using simple surveys. During the study, patients in the test group were asked to score their pain before each pill intake.  Figure 6(a) shows the survey's touch screen with a Numeric Rating Scale (NRS) [17]. The scale rates pain levels at rest and during movement.  Figure 6(b) shows representative pain score data obtained by the PCoA Acute for one selected patient. Pain score data analysis demonstrated that patients using the PCoA Acute reported significantly less pain, both at rest and in movement, from the first postoperative (PO) day to the second (Figure 6(c)). The mean rating of rest pain was 4.83 on the first PO day. This reduced to 3.22 on the second PO day (a 33.56% reduction, *P* value = 0.0058). The mean rating of movement pain was 6.38 on the first PO day and reduced to 4.25 on the second (a 28% reduction, *P* value = 0.0012). Both pain scores' reduction was statistically significant.

### 3.5. Usability of the PCoA Acute

**Figure 7:**
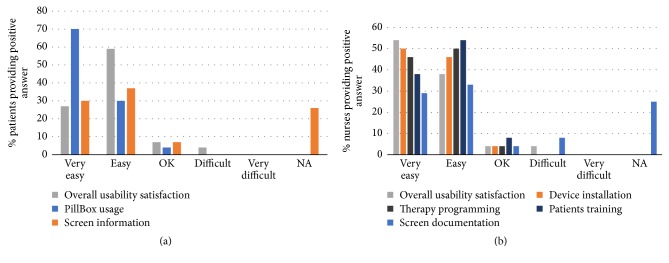
Patients (a) and medical staff (b) report ease of care and usability of PCoA Acute. Data was obtained from the questionnaires.

Usability and ease of care (EOC) of PCoA Acute were evaluated by questionnaires completed by patients and medical staff.  Figure 7 demonstrates ease of use and satisfaction of patients and medical staff with the PCoA Acute. All patients (100%) found the PillBox very easy (70%) or easy (30%) to use. Almost 90% of medical staff indicated that the device's installation, setup, and handling were easy to operate. Screen use was easy to use for 60% of patients and medical staff. Over 90% of patients and medical staff indicated their overall satisfaction with the device's use and marked the device as “very easy” or “easy” to use. All participants stated that they would recommend the use of PCoA Acute to their colleagues (data not shown). Overall, these results demonstrate high usability and overall satisfaction from the PCoA Acute as an oral PCA device for hospitalized patients suffering from postoperative pain.

## 4. Discussion

The major findings of this pilot study were as follows:

1. PCoA Acute is safe and effective as an oral PCA for hospitalized patients who require pain therapy.
2. PCoA Acute is easy to use and well accepted by patients and medical staff.
3. Use of PCoA Acute is valuable for patients and medical staff: nurse time is significantly saved compared to conventional procedure. Pill intake occasions are increased, indicating enhanced patient's adherence to their therapy regimen. Patient's pain is reduced towards the 2nd PO day indicating positive clinical outcome.

The mean time of pain medication provision by nurse upon patient's request (the control group) was found to be 8.58 minutes. Nurses were informed that the time of pill provision is being measured. This awareness certainly contributed to their motivation to shorten the measured time. These results are in agreement with a previous study indicating 10.9 minutes as average nursing time required to provide oral opioids in a postoperative orthopedic nursing unit [18]. By contrast, the mean time required for pill intake by patient using PCoA Acute was 1.17 minutes, an 86% reduction compared to current practice. These results indicate that implementation of PCoA Acute in the hospital setting may save a considerable amount of valuable nursing time.

Interestingly, and unexpectedly, the study results further showed that patients using PCoA Acute obtained more pain medications, according to their therapy regimen, relative to patients of the control group. The mean pill number obtained by a patient in the test group was 5, compared to 1.67 pills in the control group. Although the parameter of pill number per patient was not part of the study outcomes, results suggest that the rapid and simple provision of pain medication by PCoA Acute can increase pill intake events (by 67%) and lead to increased patient's compliance with their therapy regimen.

Notably, differences between the control group and the test group were statistically significant for both duration of pill administration and number of pills taken by patients. Despite the small size of groups enrolled for this pilot study, statistical significance was achieved for all medical centers and also within each medical center. The only exception was number of pills intake at MC-C, in which the control group took more pills compared to the test group (37 versus 31 pills, resp.). A possible explanation is that patients at MC-C underwent orthopedic operations. This procedure is highly painful and specific instructions are given for extra pain medication. Moreover, the department was relatively highly staffed with nurses, located centrally. Nevertheless, the time required for pill administration by nurse in MC-C was significantly longer relative to the use of PCoA Acute (8.64 min for nurse administration versus 1.55 min for PCoA Acute), as was found for all centers.

Nurse-administered pain medication is a burdensome process and requires direct interaction between patient and nurse as well as nurse availability for the patient at the right time. Moreover, the nurse is responsible for assessment and management of the patient's pain. Interruption to this process may occur for different reasons; for example, patient feels uncomfortable to distract a busy nurse; nurse is not available or delays medication provision. By contrast, use of PCoA Acute enables the patient to control his pain medication consumption. This results in increased number of pills intake by the patient during his hospitalization period. Studies conducted in various clinical settings have consistently shown that nurses tend to underestimate patients' pain and undermedicate patients for their pain [3]. They usually administer analgesics at the lower end of possible doses even when patients' pain is not relieved by these doses [4].

Rosati et al. conducted a pilot clinical study to evaluate the functionality and usability of an oral PCA device for oncology patients. Patients reported that use of the device provided better pain control, since it allowed them to receive medication directly without delay. Moreover, all patients preferred using the device to calling a nurse for each dose of as-needed medication. In addition, most nurses reported that the device saved their time and that patients' pain appeared to be better controlled when the device was used [19]. These results are in agreement with the results of the present study, demonstrating that use of oral PCA device for pain management in the hospital setting is effective and beneficial for both patients and nurses.

The 2016 guideline, for the management of postoperative pain, from the American Pain Society and the American Society of Regional Anesthesia and Pain Medicine, strongly recommends oral over IV administration of opioids for postoperative analgesia, in patients who can use the oral route. IV PCA is only necessary in hospitalized patients with an ileus, aspiration risk, or after surgical procedures that affect the ability to take medications orally [11].

Comparable oral PCA devices have been introduced to hospitalized patients with postoperative pain medication; Sufentanil Sublingual Tablet System (SSTS, AcelRx Pharmaceuticals) uses Sufentanil tablets for sublingual transmucosal drug uptake [20]. SSTS was compared to IV PCA system, for the management of acute postoperative pain, in a randomized, open-label, study. Results show that ease of care and satisfaction scores were higher with SSTS compared to IV PCA, while safety and efficacy were similar [21]. These results indicate that oral PCA can successfully replace IV PCA. However, SSTS uses only Sufentanil as an analgesic drug, while PCoA Acute is flexible and can be adjusted to most oral analgesics routinely used in the clinic.

MOD® (Medication on Demand) is another oral PCA device (Avancen MOD Corporation). A clinical study evaluated pain management in patients after total knee arthroplasty, compared between MOD and usual care of nurse-provided pain medications. It was found that device patients had significantly better pain scores than the usual care group. Moreover, all measured functional parameters, including general activity, mood, sleep, and appetite, were significantly better in the device group than in the usual care group [12].

The fast administration of pain medications, the high number of consumed pills, and the pain scores reduction for patients using the PCoA Acute suggest that pain control is improved by using PCoA Acute and that the device can be successfully used for real time pain evaluation of postoperative hospitalized patients.

### 4.1. Study Limitations

Limitations of the study included the heterogeneous small groups size, different surgical procedures, and differing use of step 1 and 3 analgesics applied at each medical center (though similar for both groups within each medical center). Statistical analysis confirmed significant difference between groups in all centers and also within the study group in each medical center. However, differences observed between control groups within medical centers were not statistically significant. More young patients aged < 65 were enrolled to the control group. The number of pills consumed by patients in the test group at MC-C was lower compared to the control group. This may be due to a different patient population, the availability of medical staff, and organization and logistics on the wards. For the control group, more patients aged below 65 years were enrolled. The study had a sequential design; the test group study was initiated only after the control study was completed in each medical center; hence, randomization, as defined, was not conducted. With regard to opioid-induced symptoms or other adverse effects of drugs, symptoms as nausea, vomiting, dizziness, fatigue, or others were not assessed because of the study's specific objectives and according the ethical approval which only allows the evaluation of a medical device but not pharmacological endpoint. Ethical approval limited us to the assessment of adverse effects only in relation to the device.

Evaluation of clinical outcome requires different study design, larger sample size, and homogeneous patient population. This therefore was not part of the study goals and will be tested in a pivotal clinical study specifically designed to meet this goal.

## 5. Conclusions

All study endpoints were achieved. 

(*1) Safety Endpoint.* The PCoA Acute system is safe under the study conditions: no severe adverse events, no pill provision during the lockout interval, no overdose, and no pill malformation.

(*2) Efficacy Endpoint.* 82% of patients in the test group completed the study and successfully used PCoA Acute to obtain their pain medication. Only rare device malfunctions were reported. The average time for pill intake was reduced by 86%, from 8.58 minutes under current practice of nurse-provided medication to 1.17 minutes with the PCoA Acute. Moreover, patients in the test group took 67% more pills (average of 5 pills/patient in the test group compared to 1.67 pills/patient in the control group). This data indicated better therapy compliance. Pain scores of patients using PCoA Acute were significantly reduced on the 2nd PO day, indicating desired clinical outcome. Almost all differences between study groups were statistically significant. 

(*3) Usability Endpoint.* The PCoA Acute was well accepted by medical staff and patients. Over 90% of study participants described it as easy to use and were satisfied with its use.

## Figures and Tables

**Table 1:** Demographics and baseline characteristics of patients participating in the study (MC: Medical Center).

Characteristic	Control group *n*%/(*n*)				Test group *n*%/(*n*)				Total: All *n*%/(*n*)
	MC-A (*n* = 20)	MC-B (*n* = 12)	MC-C (*n* = 11)	Total (*n* = 43)	MC-A (*n* = 9)	MC-B (*n* = 9)	MC-C (*n* = 9)	Total (*n* = 27)	Total: all (*n* = 70)
Age (years)									
<65	85 (17)	67 (8)	73 (8)	76 (33)	55 (5)	33 (3)	67 (6)	52 (14)	67 (47)
>65	15 (3)	33 (4)	27 (3)	24 (10)	45 (4)	67 (6)	33 (3)	48 (13)	33 (23)
Sex									
Male	35 (7)	75 (9)	45 (5)	49 (21)	45 (4)	45 (4)	45 (4)	44 (12)	47 (33)
Female	65 (13)	25 (3)	55 (6)	51 (22)	55 (5)	55 (5)	55 (5)	56 (15)	53 (37)
Type of surgery									
ENT	95 (19)			44 (19)	100 (9)			33 (9)	40 (28)
Gynecology	5 (1)			2 (1)					2 (2)
Orthopedics			100 (11)	26 (11)			100 (9)	33 (9)	28 (20)
Thorax		100 (12)		28 (12)		100 (9)		33 (9)	30 (21)
Pain therapy medication									
Oxycodone 5 mg	100 (20)			47 (20)	100 (9)			33 (9)	41 (29)
Oxycodone 10 mg		100 (12)		28 (12)		100 (9)		33 (9)	30 (21)
Morphine 10 mg			100 (11)	25 (11)			100 (9)	33 (9)	29 (20)

**Table 2:** Summary of actions performed by patients in the test group, to obtain pain medication by PCoA Acute. Data was obtained from PCoA Acute log files.

Action	Total pills	% patients performed the action	Average pills per patient
Pill intake	135	100	5
Pill request during lockout interval	27	60	0.4
Extra pill (by nurse)	10	29	1

**Table 3:** Analysis of pill intake time (minutes) by group and by medical center. Analysis was done by nonparametric Wilcoxon-Mann–Whitney method. Analysis of variance (ANOVA) was applied for analyzing the difference in the study outcomes between study centers within study groups (MC: Medical Center).

Parameter	All centers		MC-A		MC-B		MC-C	
Group	Control	Test	Control	Test	Control	Test	Control	Test
Number of total pills	75	135	21	55	17	49	37	31
Number of patients	43	27	20	9	12	9	11	9
*Parameter (minutes)*								
*Mean*	**8.58**	**1.17**	8.61	0.89	8.38	1.25	8.64	1.55
Std	8.04	0.55	5.92	0.40	13.66	0.43	5.50	0.68
StdErr	0.93	0.05	1.29	0.05	3.31	0.06	0.90	0.12
Min	2.00	0.38	2.42	0.38	2.00	0.74	2.52	1.03
Median	6.33	1.05	7.08	0.86	4.37	1.29	6.75	1.33
Max	60.00	3.77	28.80	1.65	60.00	2.62	28.10	3.77
*P* value (Wilcoxon test)	*P* < 0.0001		*P* < 0.0001		*P* < 0.0001		*P* < 0.0001	
*P* value for difference between the centers by group (ANOVA)			*Control:* *P* = 0.9937 *Study:* *P* = 0.0003					

**Table 4:** Analysis of total pills administered by group. Analysis was done by nonparametric Wilcoxon-Mann–Whitney. Analysis of variance (ANOVA model) was applied for analyzing the difference in the study outcomes between study centers within study groups (MC: Medical Center).

Parameter	All centers		MC-A		MC-B		MC-C	
Group	Control	Test	Control	Test	Control	Test	Control	Test
Number of total pills	75	135	21	55	17	49	37	31
Number of patients	43	27	20	9	12	9	11	9
*Parameter (number of pills/ patient)*								
Mean	1.67	5.00	0.95	6.11	1.42	5.44	3.27	3.44
Std	2.28	2.42	1.96	2.71	1.78	1.94	2.65	1.88
Min	0.00	1.00	0.00	3.00	0.00	2.00	0.00	1.00
Median	1.00	6.00	0.00	6.00	0.50	6.00	3.00	4.00
Max	8.00	12.00	8.00	12.00	5.00	7.00	7.00	7.00
Lower 95% confidence limit	0.97	4.04	0.03	4.03	0.28	3.95	1.49	2.00
Upper 95% confidence limit	2.37	5.96	1.87	8.20	2.55	6.94	5.05	4.89
*P* value (Wilcoxon test)	<0.0001		*P* = 0.0005		*P* = 0.0037		*P* = 0.7915	
*P* value for difference between the centers by group (ANOVA)			*Control:* *P* = 0.0184 *Study:* *P* = 0.0448					

## Abbreviations

PCA: Patient-controlled analgesia
IV: Intravenous
PCEA: Patient-controlled epidural analgesia
PCoA: Patient-controlled oral analgesics
RFID: Radio frequency identification
PO: Postoperative
ENT: Ear, Nose, and Throat
MC: Medical Center
Std: Standard deviation
StdErr: Standard error
NRS: Numerical rating scale
EOC: Ease of care.
